# Bioinspired Superhydrophobic Nanocoating Based on Polydopamine and Nanodiamonds to Mitigate Bacterial Attachment to Polyvinyl Chloride Surfaces in Food Industry Environments

**DOI:** 10.1021/acs.iecr.3c04230

**Published:** 2024-03-27

**Authors:** William DeFlorio, Abdulla Zaza, Yashwanth Arcot, Younjin Min, Alejandro Castillo, Matthew Taylor, Luis Cisneros-Zevallos, Mustafa E. S. Akbulut

**Affiliations:** † Artie McFerrin Department of Chemical Engineering, Texas A&M University, College Station, Texas 77843, United States; ‡ Department of Chemical Engineering, Texas A&M University at Qatar, Doha 23874, Qatar; § Depart of Chemical and Environmental Engineering, University of California, Riverside, California 92521, United States; ∥ Department of Food Science and Technology, Texas A&M University, College Station, Texas 77843, United States; ⊥ Department of Animal Science, Texas A&M University, College Station, Texas 77843, United States; # Department of Horticultural Sciences, Texas A&M University, College Station, Texas 77843, United States

## Abstract

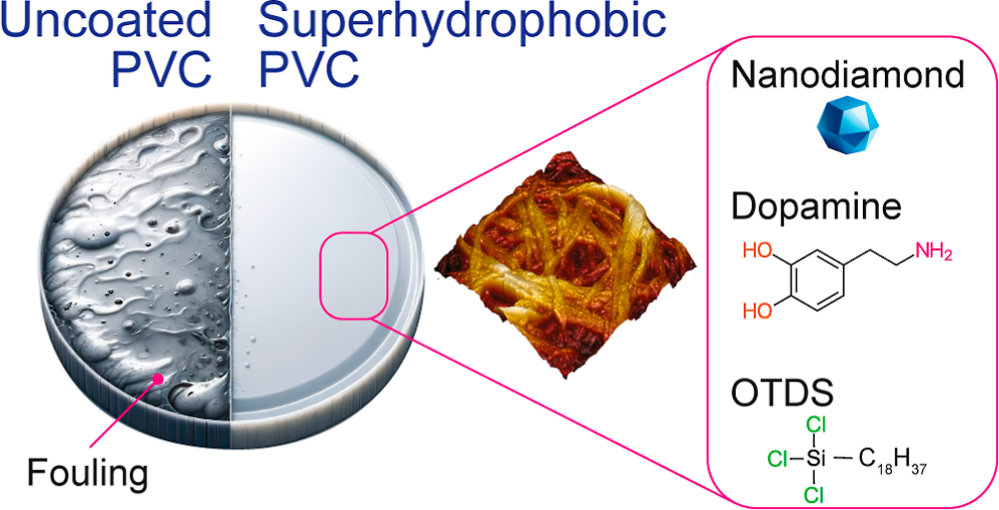

Polyvinyl chloride (PVC) is commonly utilized as a food-contact surface by the food industry for processing and storage purposes due to its durability, ease of fabrication, and cost-effectiveness. Herein, we report a composite coating for the superhydrophobization of PVC without the use of polyfluoroalkyl chemistry. This coating rendered the PVC superhydrophobic, exhibiting a static water contact angle of 151.9 ± 0.7° and a contact angle hysteresis of only 3.1 ± 1.0°. The structure of this composite coating, consisting of polydopamine, nanodiamonds, and an alkyl silane, was investigated by utilizing both scanning electron microscopy and atomic force microscopy. Surface chemistry was probed using attenuated total reflectance-Fourier transform infrared, and the surface wetting behavior was thoroughly characterized using both static and dynamic water contact angle measurements. It was demonstrated that the superhydrophobic PVC was cleanable using a food-grade surfactant, becoming wet in contact with high concentration surfactant solutions, but regaining its nonwetting property upon rinsing with water. It was demonstrated that the coating produced a 2.1 ± 0.1 log_10_ reduction (99.2%) in the number of *Escherichia coli* O157:H7 cells and a 2.2 ± 0.1 log_10_ reduction (99.3%) in the number of *Salmonella enterica* Typhimurium cells that were able to adsorb onto PVC surfaces over a 24 h period. The use of this fluorine-free superhydrophobic coating on PVC equipment, such as conveyor belts within food production facilities, may help to mitigate bacterial cross-contamination and curb the spread of foodborne illnesses.

## 1 Introduction

World Health Organization (WHO) estimates have stated that as many as 600 million cases of illness each year result from the consumption of tainted food.^1^ A major fraction of foodborne illnesses worldwide are caused by bacterial pathogens.^2^ The United States Centers for Disease Control and Prevention (CDC) estimates that there are 48 million foodborne illnesses in the U.S. each year.^3^ It has been suggested by a study of major pathogens in the U.S. that 39% of foodborne illnesses and 64% of the resulting deaths can be ascribed to bacterial origins.^4^ It has been further suggested that cross-contamination of food in the supply chain can be a contributing factor in large scale illness outbreaks.^5,6^ As many as 29% of all food safety loss incidents are the result of microbiological pathogen cross-contamination of food preparation surfaces.^7^

Several surface modification and coating technologies have been developed to combat bacterial biofilm formation on abiotic surfaces.^8−12^ Generally, these technologies can be grouped into two categories.^13,14^ The first group, consisting of antibacterial coatings, contain materials such as peptides,^15^ metal nanoparticles,^16,17^ and charged polymers^18,19^ that inactivate bacterial cells that come into close proximity of the coated surfaces. Antibacterial efficacy can also be achieved through the strategic design of surface texture and topography.^20,21^ Specifically, the incorporation of sharp nanofeatures has been reported to inactivate bacteria by inducing mechanical stresses and ruptures in bacterial cell membranes.^22−26^ Namely, these mechano-bactericidal actions, mediated by precisely engineered sharp nanostructures, provide a physical means of bacterial inactivation.^23^ The second group relies on coating strategies to prevent bacterial attachment on surfaces rather than inactivating bacteria after/during attachment. To this end, hydration/osmotic repulsion-based coatings^27−29^ and superhydrophobic coatings^30−32^ have been developed by various research groups to prevent bacterial adhesion.

Among the various categories of coatings against bacterial contamination, inert superhydrophobic coatings are particularly useful as food-contact surfaces for a couple of reasons. First, superhydrophobic coatings often reduce the amount of viscous, aqueous food residue that is able to cling to food packaging surfaces, potentially mitigating food wastage.^33,34^ Second, in food industry settings, many food-contact surfaces require daily sanitation and cleaning procedures.^35^ With the use of superhydrophobic coatings, water usage for such activities can be reduced. Because of these advantages, superhydrophobic surfaces and coatings have received extensive research interest within the agricultural and food production sectors.^36−42^ Some coating preparations, in addition to being superhydrophobic, have included silver nanoparticles for enhanced effectiveness in resisting bacterial colonization.^43^ Some superhydrophobic surfaces were designed with ultrasharp textures to achieve dual-functionality of bacterial anticontact and mechanical/perforation/impaling antibacterial action.^44,45^ Many superhydrophobic coatings have been developed with the use of polyfluoroalkyl substances in order to possess the requisite low surface energy. Due to concerns of the negative impacts of fluorinated substances on human and environmental health, many coatings have recently been focused on the development strategies which do not contain fluorine.^46−48^ The need to eliminate fluorine from superhydrophobic coatings specifically within the context of food contact applications has been discussed extensively in published literature reviews.^36,49^ The physics behind superhydrophobic coatings and their potential role in the food processing and packaging industries have likewise been the subjects of recent publications.^41,50,51^

Polyvinyl chloride (PVC) has seen extensive use in food-contact applications including conveyor belts, rollers, piping, tubing, gaskets, gloves, countertops, and packaging.^52−56^ PVC conveyors have been the topic of extensive study. The viability of pathogenic microorganisms on and transfer of such organisms to and from PVC conveyor belts have been studied extensively.^57−60^ Some research has suggested that PVC conveyor belts may be more difficult to effectively clean and sanitize than other food-contact surfaces such as stainless-steel.^61^ The presence of two pathogenic Gram-negative rod-shaped bacteria, *Escherichia coli* (*E. coli*) and *Salmonella enterica* serovar Typhimurium (*S. enterica*), on PVC conveyor belts and rollers in food manufacturing facilities has received a noteworthy amount of research effort.^62−64^

Superhydrophobic PVC was produced in several ways. While PVC itself is typically slightly hydrophobic, with a static water contact angle (SWCA) of approximately 90°, the wetting properties can be altered slightly by adding different plasticizers.^65^ To render PVC superhydrophobic, both a low surface energy and micro-/nanoscale roughness are required. Due to the inherently low surface energy of PVC, superhydrophobic films of this material have been successfully fabricated. This has been achieved by introducing microscale porosity through solvent/nonsolvent phase separation processes.^66−68^ Requisite roughness has also been achieved via the introduction of silica nanoparticles^69^ or from casting PVC in a mold formed from a lotus leaf.^70^ Additionally composite coatings have been prepared on PVC substrates from materials including stearic acid, carnauba wax, and titania nanocrystals.^71,72^

Herein is reported the preparation and characterization of a fluorine-free composite coating of polydopamine (PDA), nanodiamonds, and alkyl ligands applied to a PVC substrate. The ability of this coating to resist fouling by both *E. coli* and *Salmonella* when contacted by both bacterial suspensions and simulated contaminated lettuce leaves is demonstrated. The ability of the coating to retain its Cassie–Baxter wetting behavior and consequent superhydrophobicity when cleaned with high concentration surfactant solutions is also demonstrated. If applied to food-contact PVC conveyor belts or rollers in an agricultural processing facility, such as in a lettuce washing and packaging line, this fluorine-free coating may serve to mitigate bacterial cross contamination and curb the impact of large-scale foodborne illness outbreaks.

## 2 Materials and Methods

### 2.1 Materials

18.20 MΩ·cm ultrapure water was generated with a Barnstead GenPure Pro water purification system equipped with ultrafiltration and UV photooxidation modules from Thermo Fisher Scientific (Waltham, MA, USA). PVC sheets 0.32 cm in thickness were obtained from SIBE-R Plastics Supply (Ocala, FL, USA). Anhydrous ethanol was obtained from Decon Laboratories (King of Prussia, PA, USA) and 100 nm diamonds were obtained from Henan Union Precision Materials (Henan, China). Dopamine hydrochloride and anhydrous hexanes were purchased from Sigma-Aldrich (St. Louis, MO, USA). 1 M tris hydrochloride buffer, sodium hydroxide, and 50% w/v aqueous glutaraldehyde were purchased from Thermo Fisher Scientific (Waltham, MA, USA). Sodium hydroxide was dissolved in 18.20 MΩ·cm ultrapure water to form 1 M aqueous solutions prior to use. *N*-octadecyl trichloro silane was purchased from Gelest (Morrisville, PA, USA). Polyoxyethylene sorbitan monolaurate (Tween 20) was obtained from TCI (Tokyo, Japan) and dissolved in 18.20 MΩ·cm water prior to use.

Trypticase soy agar (TSA) and trypticase soy broth (TSB) powders were obtained from Becton, Dickinson and Co. (Franklin Lakes, NJ, USA). Phosphate-buffered saline (PBS) powder was obtained from VWR International (Radnor, PA, USA). These powders were dissolved in DI water at the appropriate concentrations, and the resulting solutions were sterilized with 121 °C steam autoclaving prior to use. Romaine lettuce was purchased from a local grocery store (College Station, TX, USA) and stored in a 4 °C refrigerator prior to use.

### 2.2 Preparation of Superhydrophobic PVC

PVC coupons were cut into 2.5 × 2.5 cm squares and cleaned with ethanol prior to use. The first coat of PDA was applied to these coupons by preparing an aqueous coating solution consisting of 0.15 w/v % dopamine hydrochloride and 1.0 w/v % 1 M tris buffer in ultrapure water, which had been titrated to a pH of 8.5 using 1 M aqueous sodium hydroxide. The PVC coupons were floated in this solution and magnetically stirred without heating under ambient conditions for 24 h. Following the application of this coating, excess was removed by rinsing in 18.20 MΩ·cm water.

100 nm diamonds, suspended 1.0 w/v % in ethanol via probe sonication, were then drop-cast onto the coupons with a 10 mL pipet. Following the evaporation of the ethanol residue, these nanodiamonds were fixed onto the PVC surfaces by sandwiching the PVC coupons between sheets of steel, applying 1.2 kPa of pressure, and placing them into an Isotemp oven manufactured by Thermo Fisher Scientific (Waltham, MA, USA). The oven was held at 45 °C for 6 h, then 75 °C for 2 h before being allowed to cool. The PVC coupons were then sonicated in ethanol for 30 min and pipet rinsed with ethanol to remove any loose nanodiamonds. Following the evaporation of the ethanol residue, a second coat of PDA was applied to the coupons using the above-described procedure.

Alkyl surface modification of the PVC coupons was achieved by preparing a 1 w/v % solution of *n*-octadecyl trichloro silane (C18) in anhydrous hexanes. The coupons were magnetically stirred in this solution for 24 h without heating under ambient conditions, then pipet rinsed with fresh hexane.

### 2.3 Microscopic Analyses

Prior to scanning electron microscopy (SEM) the coupons were coated with 15 nm of Pt/Pd using a Cressington 208HR sputter coater from Cressington Scientific Instruments (Watford, UK) to increase electrical conductivity as described elsewhere.^40,73^ A JEOL JSF-7500F FE-SEM (RRID:SCR_022202) microscope manufactured by JEOL (Tokyo, Japan) was utilized. Secondary electron images were taken at a working distance of 15 mm. For measurement of coating thickness, ImageJ software ( National Institute of Health, Bethesda, MD, USA) was used to measure the coating cross-section.

Atomic force microscopy (AFM) was conducted using a Dimension Icon AFM manufactured by Bruker (Billerica, MA, USA) in tapping mode (RRID:SCR_022202). Data analysis was performed using Nanoscope 1.8 software, also from Bruker (Billerica, MA, USA).

### 2.4 Surface Wetting Behavior Analyses

Static and dynamic water contact angles were measured using an OCA11 goniometer and tensiometer manufactured by DataPhysics Instruments (Charlotte, NC, USA). The sessile drop technique was used. For static measurements, 5 μL droplets of 18.20 MΩ·cm water were placed onto the surfaces, and a Laplace–Young fitting was used to measure the SWCAs from the drop profiles. Dynamic contact angles were measured by placing 5 μL droplets onto the surfaces and leaving the needle within the droplet. 15 μL of water was then added to each of these captive droplets at 0.5 μL/s, and a tangent leaning algorithm was used to measure the advancing contact angles (ACAs) from the droplet profiles. After a 60 s hold to re-establish equilibrium, water was then retracted from each droplet at 0.5 μL/s and the same data processing procedure was used to measure the receding contact angles (RCAs).

The sliding angle of the superhydrophobic PVC was measured by placing 20 μL droplets of 18.20 MΩ·cm droplets of water onto the superhydrophobic surfaces. The surfaces were manually tilted, and videos were recorded using a Moticam 1000 1.3 M pixel camera made by Motic (Xiamen, China). ImageJ software from the National Institute of Health (Bethesda, MD, USA) was then used to measure the angle at which the droplet started to roll in each video.

To explore the effects of surfactant on surface wetting behavior, solutions of polyoxyethylene sorbitan monolaurate (Tween 20) in 18.20 M MΩ·cm water were prepared ranging in concentration from 0 to 50 g/L. 5 μL droplets of these solutions were placed onto the superhydrophobic PVC surface, and static sessile drop profile pictures were captured using the Moticam camera. The B-spline type DropSnake plugin^125^ in ImageJ was used to measure the contact angles. Superhydrophobic PVC was then stirred magnetically in a 50 g/L solution of Tween 20 for 1 h and was observed to be fully wetted. The surfactant laden PVC was then stirred magnetically in 18.20 MΩ·cm water for 3.5 h, and the static contact angle was remeasured with 5 μL droplets of 18.20 MΩ·cm water.

### 2.5 ATR-FTIR Analysis

Surface composition of the superhydrophobic PVC and coupons during the intermediate stages of production was characterized with attenuated total reflectance Fourier transform infrared spectroscopy (ATR-FTIR). Spectra were recorded using a Nicolet iS5 FTIR spectrometer and analyzed using Omnic 9.2.86 software (both Thermo Fisher Scientific, Waltham, MA). For comparison purposes, solid PDA and silane condensate residues were isolated from the PDA and silane coating solutions. These were subjected to the same ATR-FTIR analysis as that of the PVC coupons.

### 2.6 Durability Testing

Durability testing was conducted by dropping 40–100 mesh silica sand from a height of 10 cm onto superhydrophobic PVC coupons, following protocols in a recent publication.^74^ Sand was dropped from a funnel in 10 g increments onto the same impact area of PVC coupons tilted at 45°. 1.5 kg of total sand was dropped onto each coupon. The SWCA of the superhydrophobic PVC was measured prior to sand abrasion using the sessile drop method with 5 μL droplets. The Moticam camera was used to take drop profile pictures, and ImageJ was utilized for measuring the contact angles. This same procedure was then used to measure the SWCA on the sand-abraded area of the PVC coupons throughout the abrasion process.

### 2.7 Bacterial Culturing

Two Gram-negative rod-shaped bacterial strains were utilized in experimentation. These were *E. coli* O157:H7 (ATCC 700728) and *S. enterica* serovar Typhimurium LT2 (ATCC 700720). These bacteria, referred to below, as “*E. coli*” and “*S. enterica*” were grown from single colony isolates and stored at 4 °C on TSA slants under mineral oil prior to use via previously published procedures.^75−77^ In brief, 10 mM and 30 g/L aqueous solutions of PBS and TSB, respectively, were prepared in DI water and sterilized via 121 °C steam autoclaving. Bacterial inocula were initially cultured in 10 mL aliquots of TSB solution by scratching sterile transfer needles on the appropriate slants and dipping them into the portions of the TSB solution. These were incubated aerobically at 37 °C for 24 h, and then, 10 μL loop transfers were made to aliquots of fresh, sterile TSB solution. These were incubated for a further 24 h in order to ensure that the stationary phase of growth was reached.

The 10 mL portions of bacteria-laden TSB solution were spun at 4000 rpm for 15 min in a Sorvall ST8 centrifuge equipped with a TX-150 swing bucket rotor, both manufactured by Thermo Fisher Scientific (Waltham, MA). Each supernatant was decanted and replaced with 10 mL of sterile PBS. Vortexing was then utilized to resuspend the bacteria. This centrifuging, decanting, and resuspension procedure was repeated twice. After the third resuspension in PBS the contents of 6 such 10 mL portions of bacteria laden PBS were combined in a sterile 250 mL flask which was then vortexed for 1 min. This procedure was carried out for each bacterial experiment, and the contents of the flask were used as the inoculum.

Serial decimal dilution in PBS and agar plating on TSA were used to measure the concentration of cells present in each inoculum. For the *E. coli* inoculum, the concentration utilized in SEM experiments was 11.9 ± 0.9 × 10^8^ cfu/mL and that utilized in simulated lettuce contamination was 12.7 ± 0.4 × 10^8^ cfu/mL. The *S. enterica* inoculum used in SEM experiments contained 21.4 ± 1.1 × 10^8^ cfu/mL cells, while that used in simulated lettuce contamination contained 15.3 ± 0.7 × 10^8^ cfu/mL.

### 2.8 Determination of Bacterial Adhesion Density

Bacterial adhesion density experiments were carried out using a previously published procedure (DeFlorio et al., 2023). Unmodified and superhydrophobic PVC coupons were floated face-down in contact with *E. coli* and *S. enterica* inocula for 24 h in covered Petri dishes under ambient conditions. Following 24 h of contact, each coupon was removed with ethanol and flame sterilized tweezers and then dipped into three containers, each with 40 mL of sterile PBS to remove any loosely clinging water film. A fixative solution containing 2.5 w/v % aqueous glutaraldehyde, the pH of which had been titrated to 7.5 with 1 M aqueous sodium hydroxide, was then applied to the coupons and held in contact for 4 h. Fresh, sterile PBS was then used to rinse away excess fixative. The samples were submerged for 10 min at a time in 20, 40, 60, and 80 w/v % aqueous ethanol and then submerged in anhydrous ethanol for 1 h. The coupons were then allowed to dry under ambient conditions for 24 h prior to microscopic examination.

Prior to SEM examination, each coupon was coated with 15 nm of Pt/Pd, as noted above. Twenty secondary electron images were then taken of each coupon at a working distance of 15 mm. For each image, a site was randomly selected on the coupon surface at the lowest possible magnification, 250×. At each location, the beam was focused and astigmatism was corrected at 2000 × magnification. Images were then captured at 1000 × magnification (120 × 90 μm field of view). The number of cells present in each image was manually counted.

### 2.9 Simulated Lettuce Contamination and Contact

Simulated lettuce contamination experiments were carried out using a previously published procedure.^39,76^ Washed romaine lettuce leaves were purchased at a local grocery store in College Station, TX and stored in a 4 °C refrigerator for less than 12 h prior to use. The top 4 cm of leaves were cut with ethanol, flame sterilized tweezers, and scissors and stored in sterile 50 mL tubes. Bacterial inoculum was then added to these tubes, submerging the leaves. The leaves were held submerged for 24 h and then removed with flame and ethanol sterilized tweezers, and each leaf section was cut in half along the vein. To remove excess water film, each leaf section was then held vertically for 1 min and laid adaxial-side-up in a Petri dish for 5 min. Leaf sections were then laid adaxially side-down onto both unmodified and superhydrophobic PVC coupons in covered Petri dishes. These were held in contact under ambient conditions on a lab bench for 4 h.

To quantify bacterial adhesion to the leaves prior to contact, some leaf sections, also held vertically then adaxial-side-up, were each dipped into three 40 mL aliquots of sterile PBS to remove any clinging water film. Each was then placed into a 50 mL centrifuge with 40 mL of sterile PBS. To detach bacteria from the leaf section surfaces and suspend in the PBS supernatant, these tubes were vortexed for 3 min and then agitated at 750 rpm for 30 min on a shaker plate. The 1.00 mL portion of each supernatant was then decimally diluted in PBS and plated on TSA to quantify culturable bacterial concentration.

To quantify the transfer of bacteria from lettuce leaves to the PVC coupons following 4 h of contact time, each PVC coupon was similarly dipped into three 40 mL aliquots of sterile PBS and placed into a fourth. As with those containing the contaminated leaves, these tubes were each agitated and the supernatant fluid from each as serially decimally diluted and plated on TSA.

### 2.10 Statistical Analyses

All of the experiments described were conducted in at least triplicate. Excel 2016 from Microsoft (Redmond, WA, USA) and MATLAB R2022b from MathWorks (Natick, MA, USA) were used to conduct analysis of variance and Tukey–Kramer posthoc tests at a significance level of α = 0.05.

## 3 Results and Discussion

### 3.1 Characterization of Surface Morphology

**Figure 1:**
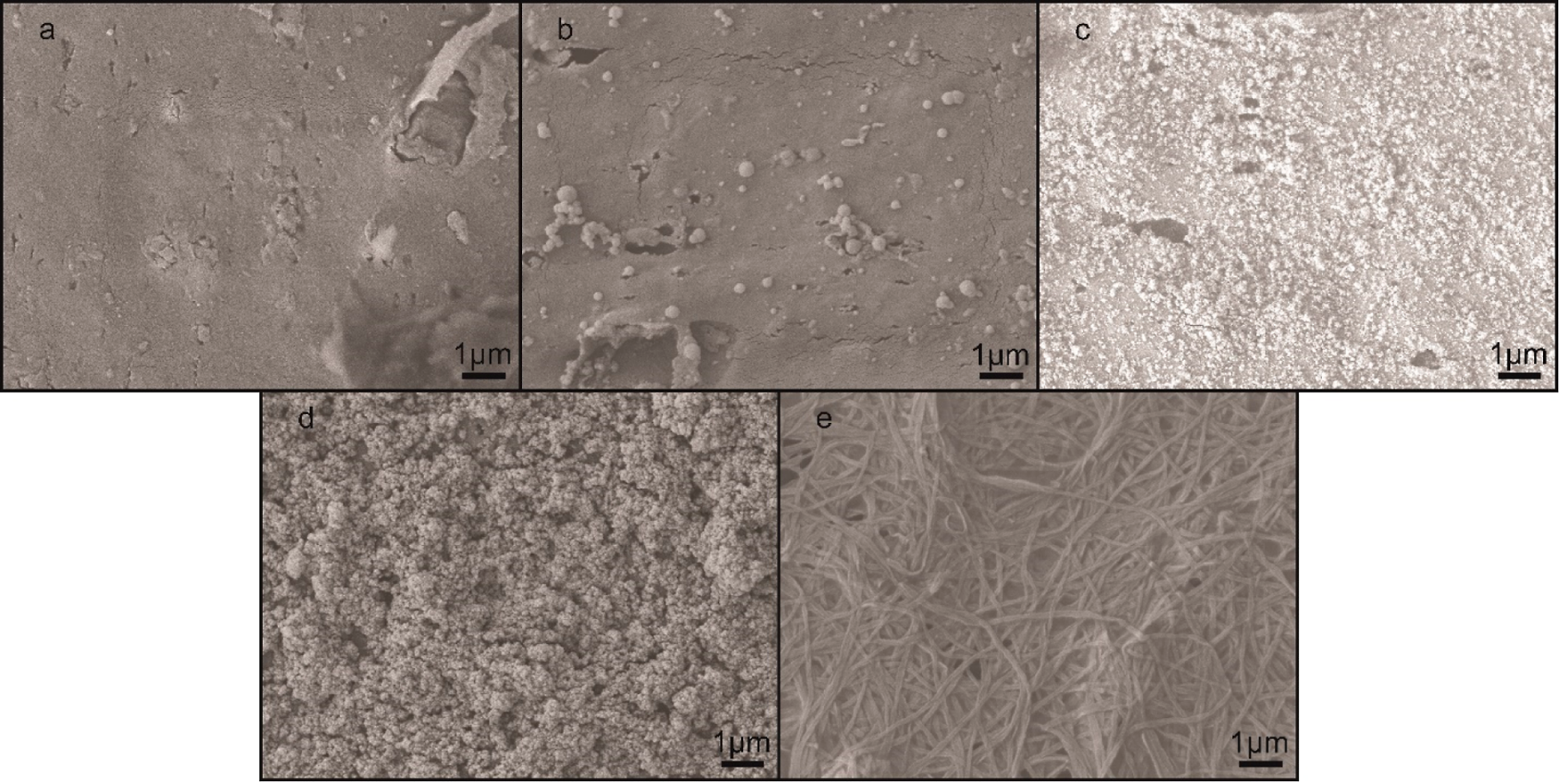
SEM (10k×) micrographs of superhydrophobic PVC coupons during each stage of production. (a) Unmodified PVC, (b) after initial coating of PDA, (c) roughened surface with embedded nanodiamonds, (d) surface after second coating of PDA, and (e) superhydrophobic PVC surface covered with alkyl ligands.

Superhydrophobicity, characterized by a SWCA of 150° or greater, requires both micro-/nanoscale roughness and a low surface energy. The fluorine-free composite coating on PVC was created via several sequential steps, which served to impart these two necessary characteristics. SEM micrographs (Figures 1a and S1a) of the unmodified PVC reveal that the plastic surface, while having some cracks and defects present, is relatively smooth. While the surface energy is low, the lack of roughness results in a SWCA of only approximately 90°.^65^ The fixation of nanodiamond particles onto surfaces in order to create the roughness required for nonwetting or superwetting behavior has some precedence.^78−81^ The advantages of nanodiamonds over other particles include their low cost, hardness, and surface chemistry decorated with oxygenated species. To aid in the drop-casting of nanodiamonds onto PVC, however, the PVC surfaces first had to be made hydrophilic in order to allow the diamond-ethanol suspension to spread evenly on the PVC coupons. This was accomplished by coating the PVC surfaces with PDA, a self-assembled monolayer rich in hydroxyl and amine groups.^82,83^ SEM examination of the PDA coated PVC in the present work (Figures 1b and S1b) showed that the coated surfaces had some PDA condensates affixed to them, but were still smooth. The hot pressing of 100 nm diamonds into the surfaces resulted in rough surfaces on in which diamond and diamond aggregates can be seen densely and uniformly covering the PVC (Figures 1c and S1c). This roughening was further enhanced by a second coating of PDA (Figures 1d and S1d), necessary for subsequent silane modification. In order to lower the surface energy of the PDA-nanodiamond composite coatings while further increasing surface roughness without the use of perfluoroalkyl substances, a final coating was applied using *n*-octadecyl trichloro silane (C_18_). This effectively covered the surface in a network of 18-carbon backbone saturated alkyl groups that were covalently bonded to the underlying material. These ligands formed an entangled network of interwoven macromolecular strands, each on the order of 10–100 nm in diameter and several microns long. This network of entangled strands (Figures 1e and S1e), comprised of alkyl groups, covered the surface in what looked like a sheet of cooked pasta noodles tangled within one another on a plate with large amounts of interstitial void space between individual strands. The dense fibrous structures enhance the roughness of the substrates and predominantly occur due to the self-assembly of long-chain organo-silanes on the substrates (leading to the formation of nanofilament-like structures).^84^ According to the literature, the initial monolayer of organosilane adsorbs physically to the PDA layer via hydrogen bonding. Subsequently, silane nanofibers grow onto the substrates via a limiting growth mechanism, where the formation of Si–O–Si covalent bonds takes place.^85^

Cross-sectional examination of the composite coating with the SEM (Figure S2) showed a 39.8 ± 2.9 μm total thickness.

**Figure 2:**
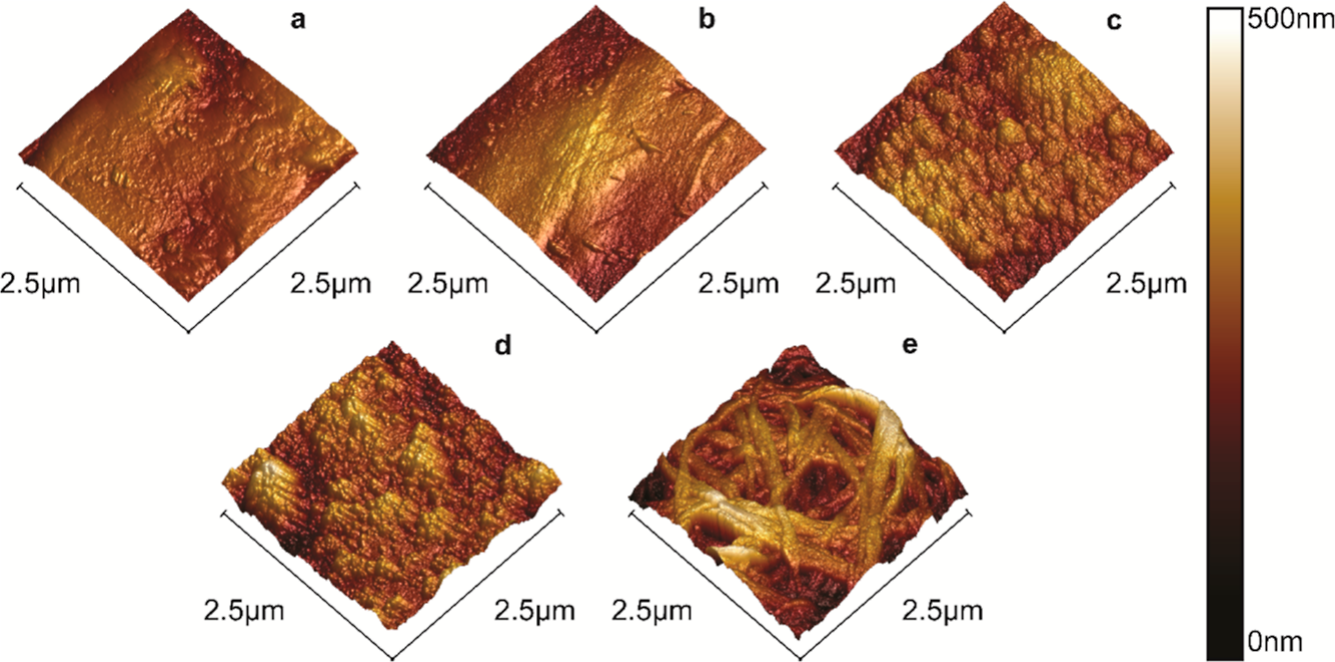
AFM images of the PVC surfaces during sequential steps in the production of the composite coating. (a) Unmodified PVC, (b) after the first PDA coating, (c) with nanodiamonds embedded, (d) after the second coating of PDA, and (e) superhydrophobic PVC surface covered with alkyl ligands.

When examined with AFM, the evolution of the surface structure (Figure 2) could be described quantitatively. The calculated roughness parameters were tabulated (Table 1). Parameters were calculated using Nanoscope 1.8 software, and formulas are included in an appendix of Supporting Information. Casual examination of the arithmetic average roughness (*R*_a_) and root-mean-squared roughness (*R*_q_) values would seem to indicate that, in spite of the obvious visual differences between the surfaces revealed by SEM and AFM, the presence of the PDA and nanodiamonds did not significantly enhance the surface roughness. *R*_a_ and *R*_q_, however, are not comprehensive descriptors of surface roughness. Another valuable parameter to consider is the roughness ratio, the ratio between actual and projected surface area. It can be observed from the roughness ratios that the presence of the PDA-nanodiamond composite resulted in a 13% increase in available surface area compared to the unmodified PVC surface. After decoration with the C18 ligands there was a 31% increase in surface area relative to unmodified PVC, reflective of the visibly rough network of intertwined structures and the interstitial voids between them.

**Table 1:** Roughness Parameters Calculated from AFM Data^a^

sample type	average roughness (*R*_a_) (nm)	root-mean-squared roughness (*R*_q_) (nm)	maximum peak height (nm)	roughness ratio
unmodified PVC	33.2 ± 2.1^A^	42.2 ± 2.6^A^	248.8 ± 14.1^A^	1.04 ± 0.00^A^
with 1 PDA coat	56.0 ± 5.4^B^	72.2 ± 6.4^B^	460.2 ± 30.8^B^	1.13 ± 0.01^B,C^
with nanodiamonds	37.4 ± 1.3^A^	47.5 ± 1.8^A^	303.0 ± 12.5^A,B^	1.10 ± 0.00^B,C^
with 2 PDA coats	45.3 ± 1.5^A,B^	58.2 ± 2.1^A,B^	379.7 ± 15.0^A,B^	1.18 ± 0.01^C^
superhydrophobic	102.1 ± 4.5^C^	131.5 ± 6.3^C^	849.7 ± 57.1^C^	1.36 ± 0.02^D^

a Superscripts indicate significant differences with the Tukey–Kramer post-hoc test (α = 0.05) within each column of the table.

The influence of bacterial roughness on bacterial adhesion, particularly when the relevant surface is not superhydrophobic and in the Wenzel wetting state has been studied extensively both empirically and via computational simulation.^86,87^ It should be noted that the unmodified PVC is in the wetted Wenzel state but that the superhydrophobic PVC is not, so implications drawn about conclusions relevant to adhesion in the Wenzel state must be considered carefully before being applied to adhesion onto nonwetting surfaces. Within the context of food-contact applications it has been suggested that average roughness greater than 0.8 μm should be avoided to minimize the potential for harborage of bacterial cells within the interstices between surface roughness features.^88^ This requirement is met by superhydrophobic PVC during all stages of production.

### 3.2 Analysis of Surface Chemistry

The PDA was coated onto PVC prior to the drop-casting of ethanol suspended nanodiamonds. The study of PDA monolayers was inspired by an interest in the underwater adhesion of marine muscles.^89^ A large body of research has been published regarding the use of PDA as a surface coating. The polymerization of dopamine is an oxidation polymerization that occurs in alkaline aqueous environments with dissolved oxygen present. The resulting polymer is richly decorated with catechol, quinone, and amine functionalities which allow it to adhere tightly to a variety of substrates, forming a self-assembled monolayer, via hydrogen bonding.^90,91^ These same hydroxyl and amine functionalities can be used as anchoring sites for subsequent attachment of ligands for further chemical modification with silanes and thiols.^82,83,92^ It should also be noted that the nanodiamonds produced by the detonation method, such as those discussed in this work, have some oxygenated lattice termination. Hydroxyl and carboxyl functional groups are created by reactions between the diamonds themselves and the cooling medium in which they are created or by subsequent purification in mineral acids.^93^

**Figure 3:**
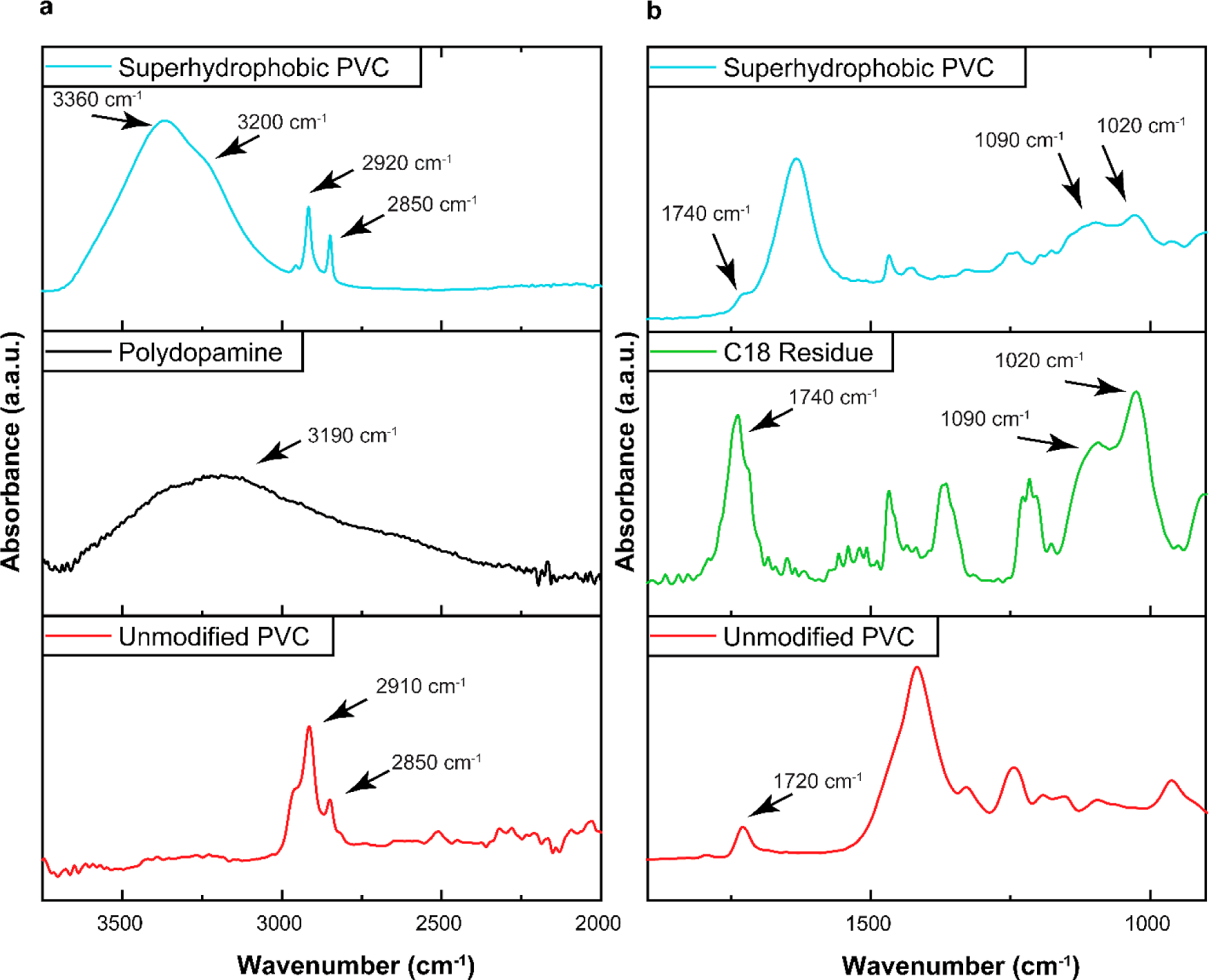
ATR-FTIR spectra of unmodified PVC, superhydrophobic PVC, and PDA or C18 alkyl silane for the wavenumber range of (a) 3600−2000 cm^−1^ and (b) 1800−900 cm^−1^.

ATR-FTIR analysis confirms the presence of PDA in the superhydrophobic PVC. The unmodified PVC spectrum (Figure 3a) shows a doublet of peaks at 2850 and 2910 cm^–1^, attributable to C–H stretching.^94^ These same peaks are very strong in the superhydrophobic PVC spectrum due to its alkyl surface chemistry. Also visible in the superhydrophobic PVC spectrum is a large, wide peak at 3360 cm^–1^ indicative of siloxane formation and hydroxyl groups.^95^ A shoulder on this wide peak is visible at 3200 cm^–1^ which can also be seen as its own distinct peak on the PDA residue spectrum. This can be attributed to the C–H or O–H stretching on the PDA aromatic rings.^96^ The presence of the C18 alkyl silane on the superhydrophobic PVC was similarly confirmed via examination of ATR-FTIR spectra (Figure 3b). The unmodified PVC spectrum has a distinct peak at 1720 cm^–1^, likely due to C=O stretching, possibly indicative of the presence of a phthalate plasticizer in the unmodified PVC.^97^ It is unclear why evidence of such carbonyl groups also exists in the C18 alkyl silane residue spectrum, but also observed in this spectrum is a pair of peaks at 1020 and 1090 cm^–1^. These peaks, also seen in the superhydrophobic PVC spectrum, may be attributable to siloxane or silanol stretching.^98^ It should once again be noted that superhydrophobic PVC, unlike many conventional superhydrophobic technologies, was prepared without the use of fluoroalkyl chemistry.

### 3.3 Surface Wetting Behavior

The ability of water droplets to wet solid substrate surfaces is a manifestation of several physical and chemical influences, including the surface micro-/nanostructure and surface energy. The common measured values used to characterize surface wetting are the SWCA, ACA, and RCA. The SWCA is a measure of how readily water droplets interact with the surface upon which they reside. The ACA has been associated with the ease with which a water droplet spreads on a surface and the RCA is indicative of adhesion forces acting to maintain contact between the water droplet and the substrate surface.^99,100^ The difference between the advancing and RCAs, or “contact angle hysteresis” (CAH), can arise from several factors, including the pinning of particles at the solid–water–vapor contact line, the adsorption of water molecules onto the substrate surface, or conformational changes of the substrate surface structure. CAH measurements of less than 5° are often indicative of uniform, isotropic surface chemistry and morphology.^101,102^

Two models are often used to describe the wetting behavior of water droplets placed onto rough, heterogeneous substrate surfaces. Wenzel type wetting is characterized by the penetration of the interstices between surface roughness asperities by liquid phase water.^103^ Superhydrophobic and nonwetting surfaces with SWCAs greater than 150°, however, are often described in terms of the Cassie–Baxter model in which the surface tension of the water droplet prevents it from effectively penetrating the interstices between surface asperities. Consequently, the water droplet resides in only partial contact with the solid substrate surface, also being supported by pockets of air trapped between the surface asperities. This results in a layer of air trapped at the interface between the solid substrate surface and any water in which it is in nominal contact.^104^ This layer of air trapped at the interface acts as a physical barrier and is largely responsible for the antifouling and other novel properties of superhydrophobic surfaces.

When water droplets are placed atop superhydrophobic surfaces, they tend to rapidly bead up and roll off of the surfaces. One measure of this behavior is the minimum sliding angle (MSA), or the angle of tilt required to move a static droplet off of a superhydrophobic surface. Low MSAs in all tilt directions are considered further indicative of Cassie–Baxter wetting and the presence of trapped interstitial air.^105,106^

**Figure 4:**
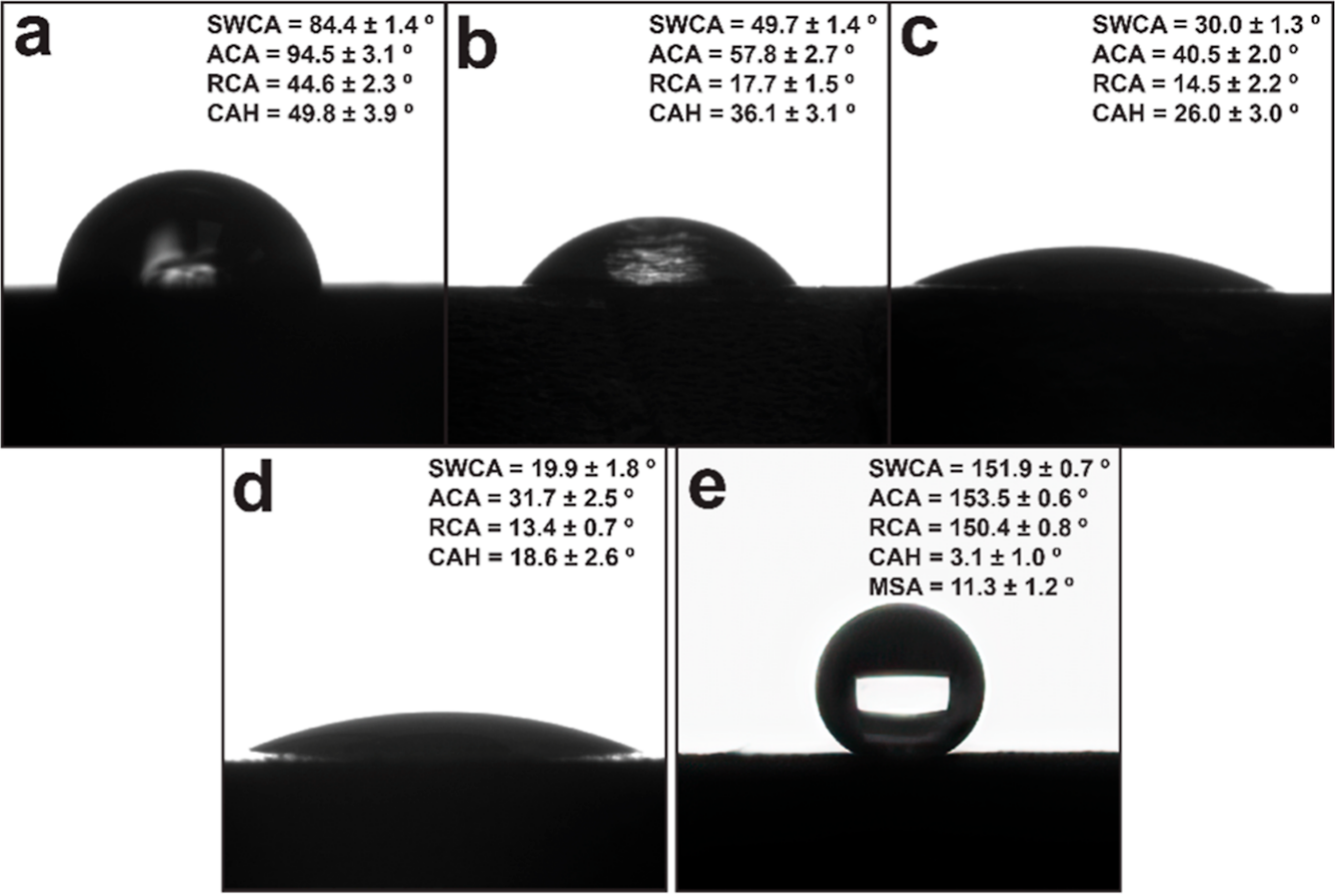
Static and dynamic water wetting behavior of the PVC coupons during each stage of production. (a) Unmodified PVC, (b) after the first PDA coating, (c) with nanodiamonds embedded, (d) after second coating of PDA, and (e) superhydrophobic PVC surface covered with alkyl ligands.

The sessile drop technique was used to evaluate the water wetting behavior of the superhydrophobic PVC surfaces during each stage of production. Unmodified PVC (Figure 4a) exhibited a SWCA of 84.4 ± 1.4°. This is slightly lower than the anticipated 90° and the discrepancy may be due to the presence of plasticizers utilized in the manufacture of the PVC (Brown et al., 2017).^65^ The subsequent applications of PDA and nanodiamonds resulted in the formation of nanorough surfaces which were richly decorated in hydroxyl, carboxyl, and amine functional groups which could participate in hydrogen bonding interactions with water molecules. This served to make the surfaces more and more hydrophilic, decreasing the SWCAs.^107^ The SWCA for PVC coated once with PDA (Figure 4b) was 49.7 ± 1.4°. The fixation of nanodiamonds onto the surfaces (Figure 4c) resulted in a further reduction of the SWCA to 30.0 ± 1.3°. A second coating of PDA atop of these immobilized NDS (Figure 4d), further decorating the surfaces with hydroxyl and amine groups, resulted in a reduction of the SWCA to 19.9 ± 1.8°. Final deposition of saturated alkyl groups onto the rough PVC surfaces served to further enhance the roughness while simultaneously lowering the surface energy. This facilitated the transition from Wenzel type wetting behavior to superhydrophobic Cassie–Baxter wetting. The superhydrophobic PVC surfaces had a SWCA of 151.9 ± 0.7° and low CAH and MSA values of 3.1 ± 1.0 and 11.3 ± 1.2°, respectively.

**Figure 5:**
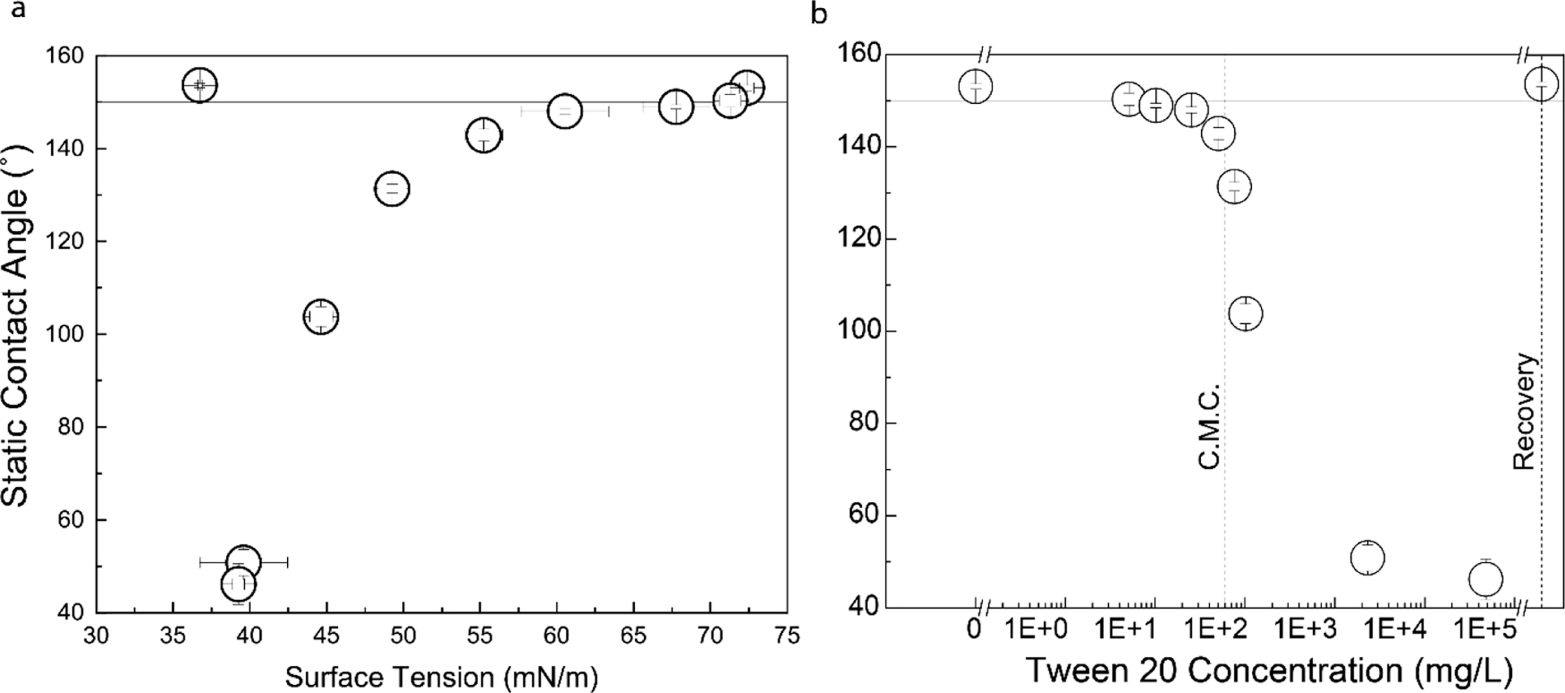
Surface wetting behavior of superhydrophobic PVC when contacted with surfactant solutions rather than water: illustrating the variation of static contact angles made by solutions on superhydrophobic PVC with (a) surface tension and (b) concentration of the surfactant solution. Error bars represent standard error.

Because Cassie–Baxter wetting phenomena are dependent on the high surface tension of the probe fluid phase, which is in contact with the solid substrate surfaces, the effect of a food grade surfactant on the modified PVC was explored. Polyoxyethylene sorbitan monolaurate, commonly known as “Tween 20″, is a food grade surfactant and strong surface wetting agent with a critical micelle concentration (CMC) of approximately 60 mg/L.^108^ Droplets of solutions with increasing concentrations of Tween 20 were placed onto superhydrophobic PVC surfaces and the SWCAs were measured (Figure 5a,b). As Tween concentration is increased in aqueous solutions up to the CMC, solution surface tension decreases. Further increasing the surfactant concentration above the CMC has minimal impact on surface tension. This behavior is typical of surfactant solutions. Because the Cassie–Baxter wetting phenomenon is dependent on the probe fluid’s high surface tension to maintain the presence of air pockets trapped at the liquid–solid interface, it might be expected that solutions of decreasing surface tension would exhibit progressively lower contact angles when placed onto a superhydrophobic surface until the surfactant concentrations exceeded the CMC, above which the contact angles would stabilize. This type of wetting behavior has been previously observed when Tween 20 solutions were placed into contact with superhydrophobic samples prepared by coating stainless-steel with a composite coating, the outermost layer of which was prepared with a fluoroalkyl silane.^39^ This type of behavior, however, was not exhibited when Tween 20 solutions were placed into contact with fluorine-free superhydrophobic PVC, the outermost layer of which was covered with alkyl groups. When Tween 20 concentrations were increased beyond the 60 mg/L CMC, static contact angles continued to decrease. This may be due to the adsorption of Tween molecules themselves onto the alkyl-decorated superhydrophobic surfaces. Each Tween 20 molecule has a hydrophobic tail essentially composed of a lauric acid molecule. Each of these tails has an 11-carbon saturated alkyl chain that allows it to take on some of the properties of a medium chain fatty acid. These chains, being similar in chemistry to the C18 alkyl chains that cover the superhydrophobic PVC surfaces, may allow the Tween 20 molecules to adsorb, effectively decorating the superhydrophobic surfaces with very hydrophilic polyethylene glycol.

After being submerged and stirred in 50 g/L aqueous solution Tween 20, it was observed that superhydrophobic PVC coupons became fully wetted and covered with a film of the Tween 20 solution that clung to their surfaces. Upon submersion and stirring to rinse in pure water, however, it was observed that any adsorbed Tween molecules or clinging film of the Tween solution was rapidly removed. The superhydrophobic PVC surfaces regained their nonwetting property even before being removed from the rinse water, as evidenced by an air gap visible to the naked eye, which formed and became trapped at the PVC-water interface. This was subsequently confirmed by SWCA measurements using the sessile drop method. The ability of the superhydrophobic PVC surfaces to be wetted by Tween 20 solutions and the subsequent recovery of their nonwetting behavior when rinsed with water seem to indicate that such superhydrophobic PVC surfaces, deployed in food production facilities, could be washed effectively without being damaged. It should be noted that sand drop tests (Figure S3) indicated that the superhydrophobic coating may be vulnerable to mechanical abrasion and periodic reapplication may be required.

### 3.4 Resistance to Bacterial Fouling

The thermodynamics and adsorption kinetics of colloid-surface interactions have historically been described using Derjaguin–Landau–Verwey–Overbeek (DLVO) type models. DLVO models describe the interaction forces acting between a surface and an approaching colloidal particle (often a planktonic bacterium) as a superimposition of van der Waals (VdW) attractions and electrostatic (ES) repulsions. VdW attraction forces decay as a function of colloid-surface separation distance (D) on the order of 1/D,^3^ while ES repulsions decay on the order of e^–D^. Numerous extensions have been made to DLVO models to account for Borne interactions, Lewis acid–base interactions, the presence of exopolymers, and various other interactions.^109−112^ It should be noted that many of the proposed models assume that there is a continuous region of aqueous media that exists between the planktonic bacterium and surface that is approaching or, in other words, that Wenzel wetting behavior is exhibited. Therefore, caution must be used when applying such models to Cassie–Baxter state superhydrophobic systems in which there are both aqueous media and trapped interstitial air pockets, which exist in the region between the bacterium and the substrate surface.

**Figure 6:**
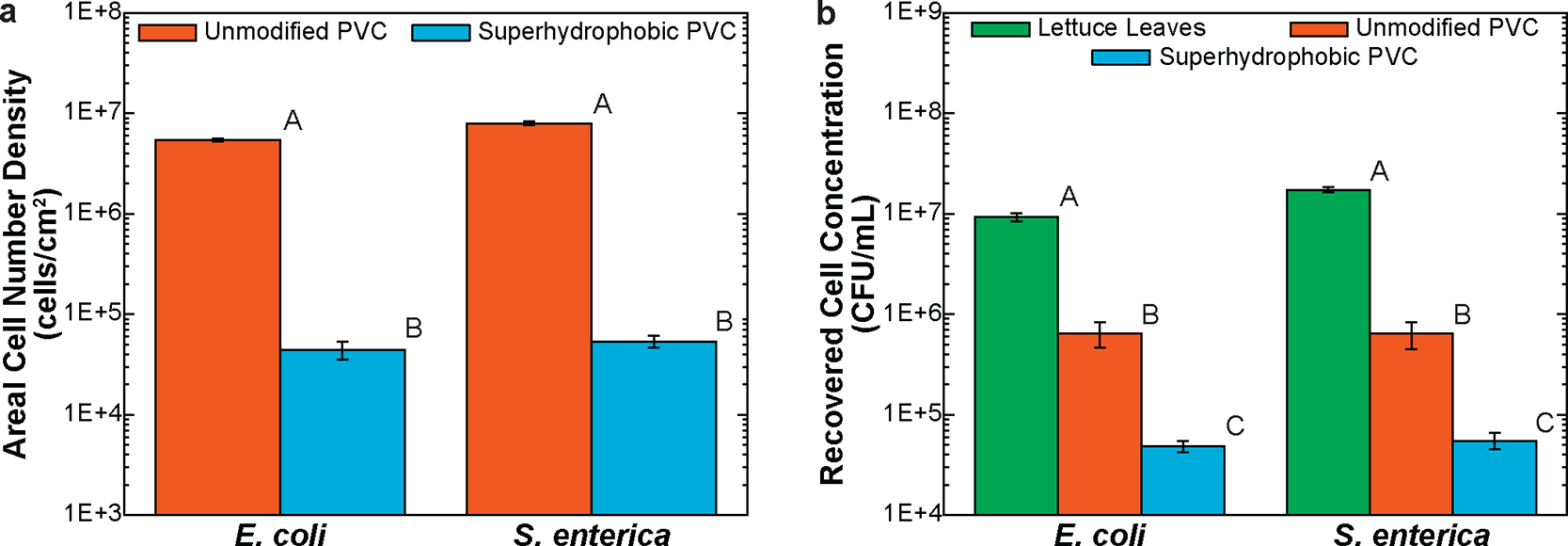
(a) Areal densities of *E. coli* and *S. enterica* cells on unmodified and superhydrophobic PVC surfaces and (b) concentrations of culturable cells recovered from simulated contaminated romaine lettuce leaves, unmodified PVC surfaces, and superhydrophobic PVC surfaces. Capital letters above bars represent the differences among average values for each bacteria type by Tukey−Kramer posthoc analysis (α = 0.05).

When planktonic bacteria approach superhydrophobic surfaces that exhibit a Cassie–Baxter wetting behavior, the layer of air trapped at the solid–liquid interface acts as a physical barrier which individual cells cannot transverse. When there is a large amount of trapped air and most of the surfaces are covered by air pockets, very little surface area remains on the superhydrophobic substrate that can be accessed by cells. This means that most cells are held at arms-length and experience longer-ranged ES repulsions but are unable to approach close enough to experience significant VdW attraction.^113,114^ Consequently, very few cells can leave the planktonic phase and adsorb onto the solid substrate to initiate biofilm formation. Previous examination of bacterial colonization onto superhydrophobic surfaces, both via SEM imaging of surfaces and via studies of recoverable, culturable cells, has consistently shown that between ∼1 and 3 orders of magnitude of bacterial colonization can be observed when superhydrophobic surfaces are in contact with aqueous innocua. This is true of both Gram-positive and Gram-negative cells in the absence of any other mechanisms of antibacterial action.^115,116^ After contact with the suspension of *E. coli* O15:H7 for 24 h, there was an areal number density of 5.4 ± 0.2 × 10^6^ cells/cm^2^ on unmodified PVC surfaces (Figure 6a). The superhydrophobic PVC surfaces, however, had only 4.4 ± 0.9 × 10^4^ cells/cm^2^. This represents a 2.1 ± 0.1 log_10_ reduction (99.2%) in the density of adhered *E. coli* O157:H7 cells. When contacted with the *S. enterica* for 24 h, there was 7.9 ± 0.3 × 10^6^ cells/cm^2^ on unmodified PVC and only 5.4 ± 0.7 × 10^4^ cells/cm^2^ on the superhydrophobic PVC. This is a 2.2 ± 0.1 log_10_ reduction (99.3%) in the density of adhered *S. enterica* cells. To put these reduction numbers into perspective, we have considered recent publications in the literature. For instance, Schumann-Muck et al.^117^ reported about 0.2 to 0.4 log_10_ reduction in the colony forming units (cfu) against *E. coli* and *Salmonella enteritidis* when silica nanoparticle-based coating was implemented on steel surfaces. Ostrov et al.^118^ tested superhydrophobic wax coatings against *Bacillus* isolates and reported up to 97–99% inhibition in bacterial attachment. Bruzaud et al.^119^ described a superhydrophobic coatings based on the electrodeposition of hydrophobic polymers (PEDOT-F4 or PEDOT-H8) on stainless steel to achieve 1.7-log reduction against of *Pseudomonas aeruginosa* compared to uncoated stainless steel. DeFlorio et al.^39^ found a 2.3 ± 0.6 log_10_ and 2.0 ± 0.9 log_10_ reductions in *E. coli* and *Listeria innocua* attachment when stainless steel was coated with nanodiamond/nickel composite layer via electroplating.

Romaine lettuce was chosen as a model food commodity for simulated cross-contamination. Both pathogenic *E. coli* and *S. enterica* can be internalized from contaminated soil and persist on lettuce leaves.^31.,126^ Both bacteria are pathogens of concern in the lettuce pre- and postharvest environment and have been linked to multistate outbreaks of foodborne diseases in the U.S. related to leafy greens.^120−122^ After 24 h submersion in *E. coli* suspension, 9.3 ± 0.8 × 10^6^ cfu/mL culturable cells were recovered from leaf surfaces (Figure 6b). After being put into contact with the adaxial side of these contaminated leaf surfaces for 4 h, 6.5 ± 1.9 × 10^5^ cfu/mL and 4.8 ± 0.6 × 10^4^ cfu/mL *E. coli* cells were recovered from the unmodified PVC and superhydrophobic PVC surfaces, respectively. These correspond to a 1.2 ± 0.1 log_10_ reduction in the number of cells recovered from the unmodified PVC surfaces compared to the contaminated leaves and a 2.3 ± 0.1 log_10_ reduction in the number of cells recovered from the superhydrophobic PVC surfaces compared to the leaves. The coating was able to produce a 1.1 ± 0.1 log_10_ reduction (92.6%) in the transfer of cells. After submersion in *S. enterica* suspension for 24 h the romaine leaf surfaces had 1.7 ± 0.1 × 10^7^ cfu/mL culturable cells. Post contact, the unmodified PVC and superhydrophobic PVC surfaces had 6.4 ± 1.9 × 10^5^ cfu/mL and 5.5 ± 1.0 × 10^4^ cfu/mL *S. enterica* cells, respectively. These represent 2.5 ± 0.1 log_10_ and 1.4 ± 0.1 log_10_ reductions in the number of culturable cells. The coating produced a 1.1 ± 0.2 log_10_ reduction (91.4%) in the transfer of *S. enterica* cells that were transferred from the contaminated romaine lettuce leaves to the PVC. As mentioned earlier, the reduction in the transfer of bacteria to superhydrophobic substrates is facilitated by trapped air stabilized by low-energy and hierarchical structures (induced by the self-assembly of hydrophobic alkyl-silane). This process greatly diminishes VdW attractions while promoting ES repulsion between negatively charged bacterial species and alkyl silane.^123^ Additionally, the preferential adhesion of planktonic cells to nutrient-rich lettuce leaves over abiotic superhydrophobic PVC could also significantly contribute to the reduction in adhesion of both Gram-positive and Gram-negative bacterial species.^124^

## 4 Conclusions

A scalable, economical method has been presented for the production of nanodiamond-based superhydrophobic coating on PVC substrates without the use of fluoroalkyl. Surface wetting behavior analysis indicated that the nonwetting property of these coatings is due to Cassie–Baxter state wetting, the consequence of a layer of air trapped at the solid–liquid interface. This superhydrophobicity, created by trapped air, was demonstrated to be effective in significantly reducing the ability of both *E. coli* O157:H7 and *S. enterica* serovar Typhimurium, both pathogens of concern, to colonize PVC surfaces. The coating was also demonstrated to reduce the number of bacterial cells transferred to PVC surfaces when it was put into contact with contaminated romaine lettuce leaves. Furthermore, it was shown that the coating can be wetted when put into contact with high concentrations of food grade surfactant but is able to recover its nonwetting property upon rinsing with water, meaning the coated surfaces are cleanable. The application of these superhydrophobic coatings to PVC used to construct conveyor belts in food processing facilities may help mitigate bacterial cross-contamination within the food production chain and curb the impacts of large-scale foodborne illness outbreaks.

## Supporting Information Available

Behavior of a droplet on the superhydrophobic PVC surface covered with alkyl ligands (MP4)
High-magnification SEM micrographs of PVC; SEM micrograph of the composite coating cross-section; and static water contact angle data of coated PVC after mechanical abrasion with sand (PDF)
