# Supplementary material for: Bioinspired Superhydrophobic Nanocoating Based on Polydopamine and Nanodiamonds to Mitigate Bacterial Attachment to Polyvinyl Chloride Surfaces in Food Industry Environments
Source: Ind Eng Chem Res. 2024 Mar 27;63(14):6235–48. doi: 10.1021/acs.iecr.3c04230 (PMC11009964; doi:10.1021/acs.iecr.3c04230)
Supplement: Supplementary file 2 — ie3c04230_si_002.pdf [file ie3c04230_si_002.pdf]

Supplemental Information

Bioinspired superhydrophobic nanocoating based on polydopamine and nanodiamonds to mitigate bacterial attachment to polyvinyl chloride surfaces in food industry environments

William DeFlorio[A], Abdulla Zaza[B], Yashwanth Arcot[A], Younjin Min[C], Alejandro Castillo[D], Matthew Taylor[E], Luis Cisneros-Zevallos[F], Mustafa E. S. Akbulut[A,*]

[A]Artie McFerrin Department of Chemical Engineering, Texas A&M University, College Station, TX, USA, 77843

[B]Department of Chemical Engineering, Texas A&M University at Qatar, Doha, Qatar, 23874

[C]Depart of Chemical and Environmental Engineering, University of California, Riverside, CA, USA, 92521

[D]Department of Food Science and Technology, Texas A&M University, College Station, TX, USA

[E]Department of Animal Science, Texas A&M University, College Station, TX, USA, 77843

[F]Department of Horticultural Sciences, Texas A&M University, College Station, TX, USA, 77843

* Corresponding Author: M. E. S. Akbulut, makbulut@tamu.edu

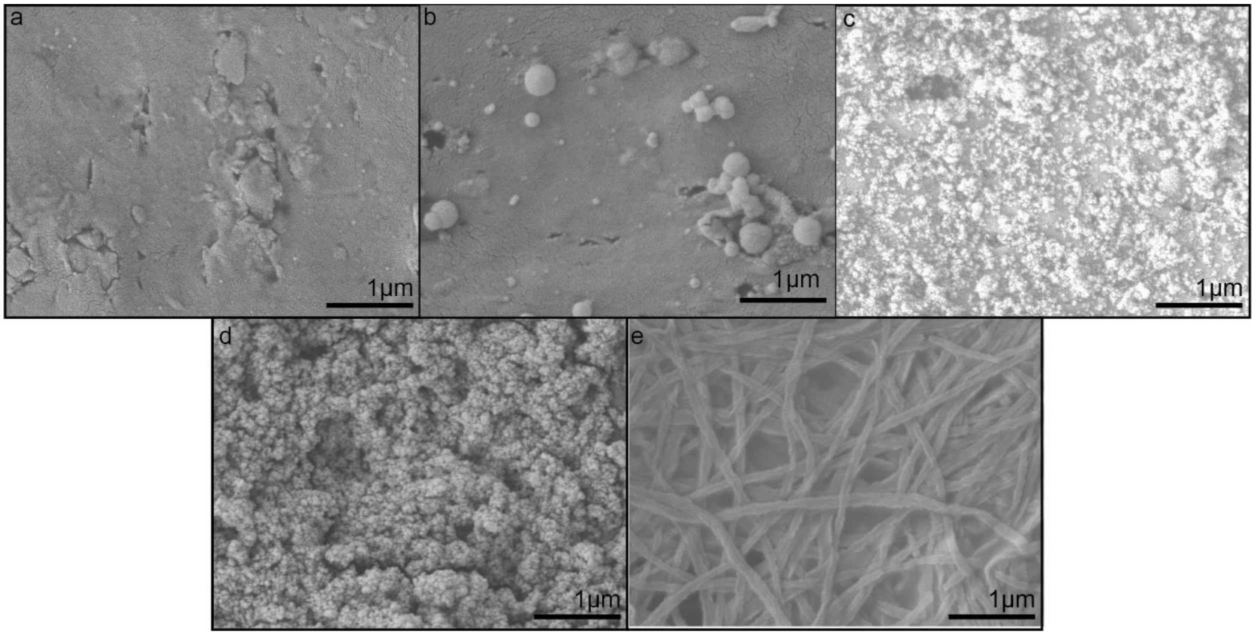

Fig. S1. Higher magnification (25k×) SEM micrographs of PVC substrates following each step in the coating procedure (a) pristine PVC, (b) after first polydopamine coat, (c) substrates embedded with nanodiamonds, (d) after second polydopamine coat, and (e) superhydrophobic PVC.

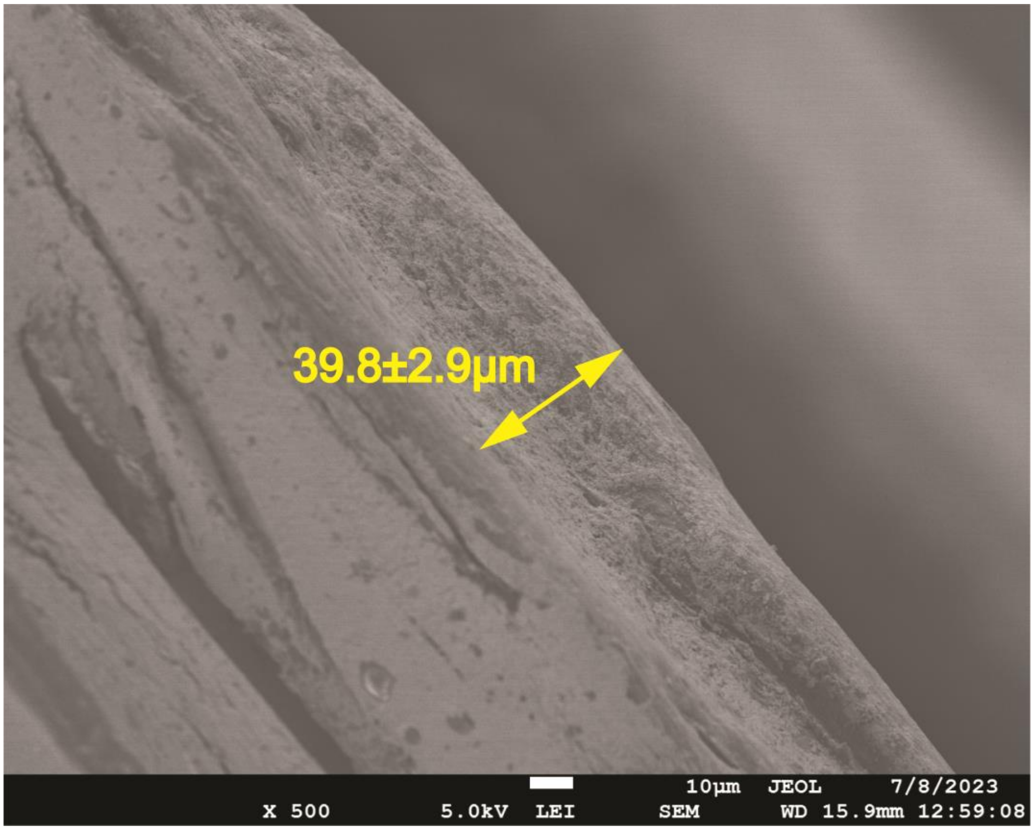

Fig. S2. SEM micrograph of composite coating cross-section.

Roughness Formulae

Average Roughness (Ra)

$$Ra = \frac{1}{L}\int_{0}^{L} |Z(x)|dx$$

Root-Mean-Squared (Rq)

$$Rq = \sqrt{\frac{1}{L}\int_{0}^{L} Z^{2}(x)dx}$$

Roughness Ratio

$$Roughness\ Ratio = \frac{actual\ surface\ area}{projected\ surface\ area}$$

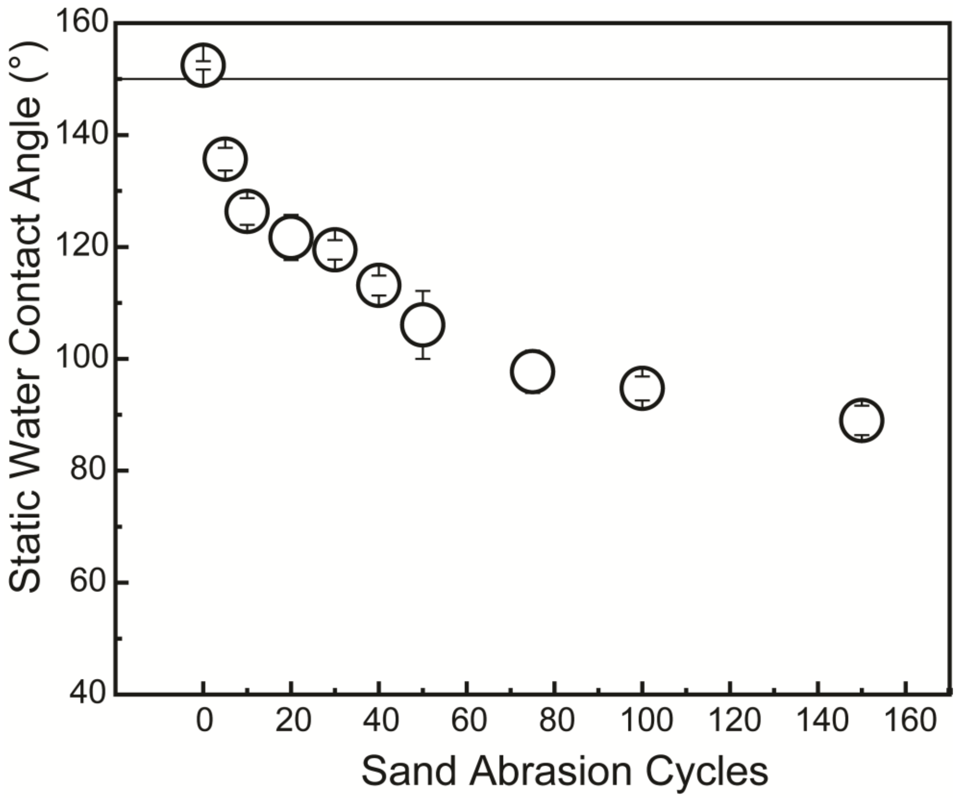

Fig. S3. Static water contact angle of coated PVC after mechanical abrasion with sand.
